# The Role of Non-Catalytic Region in Determining the Difference in Efficiency Between Two Cellobiohydrolases Revealed Through a Genetic Approach

**DOI:** 10.3390/jof11070536

**Published:** 2025-07-18

**Authors:** Xinyuan Yan, Pankajkumar Ramdas Waghmare, Xiaoli Meng, Jianhui Zhang, Shaoming Ding, Yu Lei, Jun Yue, Guodong Liu

**Affiliations:** 1 State Key Laboratory of Microbial Technology, Shandong University, Qingdao 266237, China; yanxy2024@163.com (X.Y.);; 2 Baiyin Sainuo Biotechnology Co., Ltd., Baiyin 730913, China; 3 Jilin Petrochemical Co., Ltd., PetroChina, Jilin 132021, China

**Keywords:** cellulase, cellobiohydrolase, fungi, *Penicillium*, *Trichoderma*

## Abstract

The cellulose-binding domain and inter-domain linker play crucial roles in the degradation of crystalline cellulose by cellulases. Although significant differences exist in the degradation efficiency of cellobiohydrolase I (CBH I) derived from different fungal sources, the relationship between this efficiency diversity and variations in the non-catalytic region remains poorly understood. In this study, we found significant differences in the length and amino acid composition of the linker region of CBH I derived from Sordariomycetes and Eurotiomycetes. By replacing the non-catalytic region of *Penicillium oxalicum* CBH I with the corresponding segment from *Trichoderma reesei*, the cellulose conversion efficiency of the extracellular enzyme system doubled under the same protein dosage, and the adsorption of CBH I onto cellulose was improved. While replacing only the cellulose-binding domain improved the degradation efficiency of the enzyme system, additional replacement of the linker region resulted in greater enhancement. Improved degradation efficiency due to non-catalytic region replacement was observed under various conditions, including higher cellulose substrate concentration, reduced cellulose crystallinity, use of pretreated straw as a substrate, and degradation at physiological temperature. These findings provide novel insights into the molecular mechanisms underlying crystalline cellulose degradation by filamentous fungi.

## 1. Introduction

Cellulose is the most abundant renewable organic resource on Earth. Many microorganisms secrete cellulose-degrading enzyme systems to break down cellulose into glucose and oligosaccharides for further utilization [1,2]. The enzymatic hydrolysis of cellulose is not only a crucial part of the carbon cycle in nature but also provides an effective approach to alleviating the resource and energy crises faced by humans [3,4]. A deeper understanding of the mechanisms of cellulose degradation by microbial enzymes will facilitate the rational engineering of cellulases to improve their degradation efficiency.

Filamentous fungi are widely used in the industrial production of cellulase. Generally, fungi secrete multiple enzymes, including cellobiohydrolases, endo-β-1,4-D-glucanases, β-glucosidases, and lytic polysaccharide monooxygenases, for the cooperative degradation of cellulose into glucose [2,5]. Activity tests on purified enzymes and studies on multi-enzyme combinations have demonstrated that cellobiohydrolase I (CBH I) is the key enzyme for the degradation of crystalline cellulose [6]. This enzyme constitutes approximately 50–70% of the total extracellular proteins in *Trichoderma reesei*, a well-known cellulase-producing fungus. Knockout of the gene encoding CBH I significantly impaired the growth of *T. reesei* on microcrystalline cellulose and markedly reduced the degradation efficiency of the secretome on microcrystalline cellulose [7].

A typical fungal CBH I consists of three segments: an *N*-terminal catalytic domain, a C-terminal cellulose-binding domain, and a linker region connecting the two domains. According to the classification by the Carbohydrate-Active enZymes (CAZy) database [8], the catalytic domain in fungal CBH I belongs to glycoside hydrolase (GH) family 7, while the cellulose-binding domain belongs to carbohydrate-binding module (CBM) family 1 (hereafter referred to as CBM1). The catalytic domain features a narrow, elongated tunnel containing the active site, which accommodates a single cellulose chain and slides along it for processive degradation [9]. CBM1 adopts a wedge-shaped structure that binds to the hydrophobic surface of crystalline cellulose [10], with three conserved aromatic amino acids on its flat surface playing a crucial role in substrate binding [11]. Additionally, CBM1 can directly disrupt the crystalline structure of cellulose [12]. The linker region is typically rich in serine and threonine residues and is highly O-glycosylated, generally considered to form a flexible, intrinsically disordered structure [13]. Although CBM1 and the linker peptide, as non-catalytic regions, do not directly participate in glycosidic bond hydrolysis, they play essential roles in the degradation of crystalline cellulose. Studies on *T. reesei* Cel7A have shown that removing CBM1 and the linker region does not affect its activity on soluble substrates but significantly reduces the enzyme’s adsorption and degradation capabilities on crystalline cellulose [14]. Partial deletion, mutation, or insertion in the linker sequence can also markedly impair CBH I’s adsorption and hydrolytic activity on crystalline cellulose [15,16]. Thus, a linker region with an appropriate length and structure may be necessary for cooperation between the catalytic domain and CBM1.

Although most CBH Is from fungal species share the typical “catalytic domain-linker-CBM1” architecture, their abilities to degrade crystalline cellulose vary significantly [17,18]. For example, Cel7C from *Aspergillus oryzae* degrades pretreated corn stover more than five times as efficiently as CBH I from *Trichoderma saturnisporum* [19]. Domain-swapping experiments have shown that sequence differences in the catalytic and non-catalytic regions contribute to the efficiency variations between CBH Is from different fungal sources [18,20]. Our previous research found that the difference in crystalline cellulose degradation efficiency between CBH Is from *T. reesei* and *Penicillium oxalicum* was determined by the non-catalytic region. Replacing the linker-CBM1 region in the “low-activity” *P. oxalicum* CBH I (Cel7A-2) with the corresponding sequence from *T. reesei* CBH I (Cel7A) can elevate its crystalline cellulose degradation capability to the latter’s level [21]. However, it remains unclear whether CBM1 or the linker peptide is specifically responsible for this difference.

In this study, we aimed to elucidate the structural determinants of the efficiency difference between *T. reesei* Cel7A and *P. oxalicum* Cel7A-2. First, we analyzed the sequence characteristics of the non-catalytic regions of the 245 fungal CBH Is. We then compared the roles of the linker and CBM1 regions of different origins in cellulose degradation using an in vivo gene replacement strategy. We also studied the effects of CBH I engineering on the growth of strains on microcrystalline cellulose. Together, these investigations establish a structure-function framework for rational cellulase enhancement.

## 2. Materials and Methods

### 2.1. Bioinformatics Analysis

The amino acid sequence of *T. reesei* Cel7A (NCBI RefSeq accession: XP_006969224.1) was used as a query to search for homologous sequences in the RefSeq database using PHI-BLAST (E-value ≤ 1 × 10^−5^). To identify sequences containing CBM1, the QCGG sequence conserved in CBM1 was used as the PHI pattern. Sequences with query coverage ≤ 80% were then removed. The remaining sequences were subjected to multiple sequence alignment using Clustal Omega (version 1.2.4) with the default parameters [22]. Finally, putative endoglucanases were removed based on the identification of CBH-specific loop sequences [23].

The sequences of the non-catalytic regions were retrieved according to conserved sequence motifs. The CBM1 sequence was defined as starting from the sixth residue upstream of the QCGG motif and extending to the C-terminus of the full-length protein sequence. The identified CBM1 sequences were aligned using Clustal Omega, and sequence conservation patterns were visualized using WebLogo (version 3.7.12) [24]. Phylogenetic reconstruction of the CBM1 sequences was performed using IQ-TREE (version 2.1.3; http://www.iqtree.org). The best-fit substitution model under the LG framework was selected using ModelFinder, followed by maximum likelihood tree inference with 1000 ultrafast bootstrap replicates [25]. The resulting tree was exported in Newick format and visualized using the Interactive Tree of Life (iTOL) online tool (version 7.2.1; https://itol.embl.de/) for graphical annotation and layout optimization [26]. The linker region was identified by locating the 8th residue downstream of the sequence aligned to the FSNIK sequence in *T. reesei* Cel7A as the start, extending to the *N*-terminal boundary of CBM1.

### 2.2. Construction of Strains

The *P. oxalicum* strain 114-2, isolated from decayed straw-covered soil, was deposited at the China General Microbiological Culture Collection Center (CGMCC) under the number CGMCC 5302 [27]. This strain was used as a parent for genetic engineering at the *cel7A-2* locus (GenBank: KB644414, 3959679–3961319) by homologous recombination. To construct the strain PPT, the 1530-bp sequence upstream of the CBM1-coding region in *cel7A-2* (as the left homologous arm), CBM1-coding region from *T. reesei cel7A*, 775-bp sequence downstream of *cel7A-2* (as the terminator), hygromycin B phosphotransferase gene *hph* as a selection marker, and 1554-bp sequence downstream of the terminator (as the right homologous arm) were fused using fusion PCR to obtain the gene replacement cassette. The cassette was transformed into *P. oxalicum* 114-2 via protoplast-mediated transformation [28]. The transformants were purified by plate streaking, and the correct mutants were identified by diagnostic PCR. Similarly, the PTT and TTT strains were constructed by replacing the linker-CBM1-coding region and the whole protein-coding region of *cel7A-2* with the counterparts of *T. reesei cel7A*, respectively. The control strain, named 114-2h, was constructed by integrating the *hph* gene downstream of *cel7A-2* without affecting the Cel7A-2 coding region. Additionally, a *cel7A-2* knockout strain, named Δ*cel7A-2*, was generated by replacing the entire coding sequence with *hph*. The primer sequences used for strain construction are listed in Table S1.

### 2.3. Cultivation

The *P. oxalicum* strains were cultivated on wheat bran liquor slants at 30 °C for 5 days for conidiation. Conidia were harvested by washing the slants with distilled water containing 0.9% (*w*/*v*) NaCl and 0.01% (*w*/*v*) Tween 80. For cellulase production, fresh conidia were inoculated into 50 mL seed medium at a final concentration of 10^6^ per ml, and the Erlenmeyer flasks were incubated in a rotary shaker at 200 rpm at 30 °C for 24 h. Then, 8 mL of the culture was inoculated into 80 mL of cellulase production medium in 500-mL Erlenmeyer flasks for continued cultivation at 30 °C for 7 days. The seed medium contained (g/L): wheat bran 20.0, peptone 10.0, glucose 10.0, (NH_4_)_2_SO_4_ 2.0, KH_2_PO_4_ 3.0, and MgSO_4_·7H_2_O 0.5. The cellulase production medium contained (g/L): wheat bran 30.0, microcrystalline cellulose 30.0, soybean cake powder 15.0, (NH_4_)_2_SO_4_ 2.0, KH_2_PO_4_ 5.0, and MgSO_4_·7H_2_O 0.5.

For cultivation with cellulose as the sole carbon source, strains 114-2, PPT, and PTT were cultivated in seed medium for 24 h. Subsequently, 4 mL of the seed culture was transferred into 500-mL Erlenmeyer flasks containing 80 mL of Vogel’s salts supplemented with 0.5% (*w*/*v*) Avicel PH-101 (Sigma-Aldrich, St. Louis, MO, USA) for cultivation at 30 °C for 96 h [29]. Avicel PH-101 consists of highly ordered cellulose Iβ crystallites with an average particle size of 50 μm, as characterized by the manufacturer.

### 2.4. Enzyme Assays and SDS-PAGE

Samples were centrifuged at 4 °C and 17,000× *g* for 10 min using an Eppendorf 5427R centrifuge with an FA-45-30-11 rotor (Eppendorf, Wesseling-Berzdorf, Germany). After centrifugation, the culture supernatants were collected and used for the analysis of enzyme activity and protein levels. Filter paper enzyme (FPase) and cellobiohydrolase activities were measured using Whatman No. 1 filter paper (Cytiva, Buckinghamshire, UK) and *p*-nitrophenyl-β-D-lactopyranoside (*p*NPL, J&K, Shanghai, China) as the substrate, respectively, as previously described [28]. One unit of enzyme activity was defined as the amount of enzyme that liberated 1 μmol of glucose equivalent or *p*-nitrophenol from the substrate per minute. The concentrations of extracellular proteins were measured using a Modified Bradford Protein Assay Kit (Sangon Biotech, Shanghai, China) with bovine gamma globulin as the standard.

For SDS-PAGE, the supernatants of the cellulase production cultures or hydrolysis reactions were supplemented with 5× sample loading buffer (GenStar, Beijing, China), boiled for 10 min, and loaded onto a 12% (*w/v*) SDS polyacrylamide separating gel for electrophoresis at 120 V for 1.0 to 1.5 h. Coomassie Blue R-250 (Sangon, Shanghai, China) was used for staining. The bands of interest were selected for intensity quantification using ImageJ (version 1.54p; https://imagej.net/ij/index.html (accessed on 25 February 2025)) [30,31]. The intensity values were normalized to that at the corresponding 0 h timepoint (set as 100%) to calculate the relative abundance of Cel7A-2 and the mutants in the supernatants.

### 2.5. Cellulose Saccharification

Ball-milled Avicel PH-101 was prepared by suspending 4.0 g of Avicel in 100 mL of deionized water in a 500 mL flask containing 200 glass beads (70 mm in diameter), followed by continuous shaking at 30 °C and 200 rpm for 7 days. X-ray diffraction experiments were performed using a SmartLab SE X-ray diffractometer (Rigaku, Tokyo, Japan) with Cu Kα radiation (λ = 1.5418 Å). The crystallinity index (CrI) was calculated from the diffraction profile using the Segal peak height method [32]. Liquid hot water (LHW)-pretreated corn stover was prepared by cutting corn stover into 2–3 cm segments, treating it with water at 190 °C for 1 h, and washing it with tap water. The pretreated material contained 56.65% glucan. Saccharification experiments were conducted in 100 mL Erlenmeyer flasks with a reaction volume of 30 mL. The system consisted of cellulosic substrates and enzymes at the indicated loadings, and citric acid-sodium citrate buffer (pH 4.8) at a final concentration of 0.05 M. The mixtures were incubated at 50 °C or 30 °C with shaking at 150 rpm in a rotary shaker. Samples from the saccharification process were centrifuged at 17,000× *g* for 10 min to collect the supernatants. For sugar analysis, the supernatants were boiled for 10 min and passed through a 0.22 μm filter. Glucose and cellobiose concentrations were measured using an LC-20AT HPLC system equipped with a refractive index detector (Shimadzu, Tokyo, Japan). The saccharides were separated using an Aminex HPX-87H column (Bio-Rad, Hercules, CA, USA) at 60 °C with 5 mM H_2_SO_4_ as the eluent at a flow rate of 0.5 mL/min. To calculate the cellulose conversion (% of maximum), correction factors of 0.9 and 0.95 were used for glucose and cellobiose, respectively, to compensate for the addition of water molecules during the hydrolysis of glycosidic bonds.

### 2.6. Measurement of Intracellular Proteins

Due to the insolubility of the carbon source Avicel, cell biomass was measured indirectly by determining the amount of intracellular proteins. Specifically, 1 mL of culture broth was centrifuged at 8000× *g* for 30 min, and then the precipitate was washed with 1 mL 0.9% (*w*/*v*) NaCl solution. The precipitate was then resuspended in 1 mL of 1 M NaOH solution and incubated at 200 rpm for 1 h at room temperature. The suspension was centrifuged at 8000× *g* for 10 min, and the protein content of the supernatant was determined using a Modified Bradford Protein Assay Kit (Sangon Biotech, China).

### 2.7. Statistical Analysis

Statistical analyses and graphical representations were performed using GraphPad Prism (version 9.0; GraphPad Software, Boston, MA, USA). For intergroup comparisons, datasets were subjected to one-way analysis of variance (ANOVA) with Tukey’s post-hoc test to evaluate statistical significance across multiple groups. Quantitative results are expressed as the mean ± standard deviation (SD) of triplicate measurements. Significance thresholds were rigorously defined as follows: ns (not significant, *p* > 0.05), * *p* < 0.05, ** *p* < 0.01, *** *p* < 0.001, and **** *p* < 0.0001.

## 3. Results and Discussion

### 3.1. Sequence Characteristics of the Non-Catalytic Regions of GH7 CBHs

**Figure 1:**
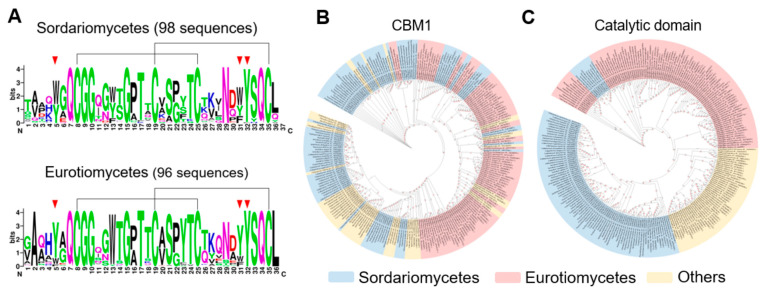
CBM1 sequences in GH7 cellobiohydrolases. (**A**) Sequence logos of CBM1s from Sordariomycetes and Eurotiomycetes. The conserved disulfide bond-forming cysteines (black lines) and cellulose-binding aromatic amino acids (red inverted triangles) are indicated. (**B**,**C**) Phylogenetic analysis of 245 CBM1s and their catalytic domains, respectively, in this study. Phylogenetic trees with high resolution are included in the Supplementary Materials (Figures S1 and S2).

Using the Cel7A sequence of *T. reesei* as a reference, we identified 245 GH7 CBHs with the catalytic domain-linker-CBM1 architecture encoded by 217 fungal genomes in the RefSeq database (Table S2). The non-catalytic regions of enzymes from two widely distributed classes, Sordariomycetes (98 sequences) and Eurotiomycetes (96 sequences), were analyzed as a key focus. Sequence alignment revealed that several positions (e.g., the four cysteines for disulfide bond formation and three aromatic amino acids directly involved in cellulose binding) are highly conserved in CBM1s (Figure 1A). No class-specific conserved amino acid residues were found in the sequences of Sordariomycetes and Eurotiomycetes. Further phylogenetic analysis of the CBM1s showed that sequences from the two classes were promiscuously grouped (Figure 1B and Figure S1, 77.0% of bootstrap values greater than 50%), confirming that the domain did not evolve through strict vertical descent. In stark contrast, the phylogeny of the catalytic domains revealed that these domains were largely clustered along taxonomic lines, clearly separating the Sordariomycetes and Eurotiomycetes (Figure 1C and Figure S2). These results suggest distinct evolutionary trajectories between CBM1 and the catalytic domain of GH7 CBHs.

**Figure 2:**
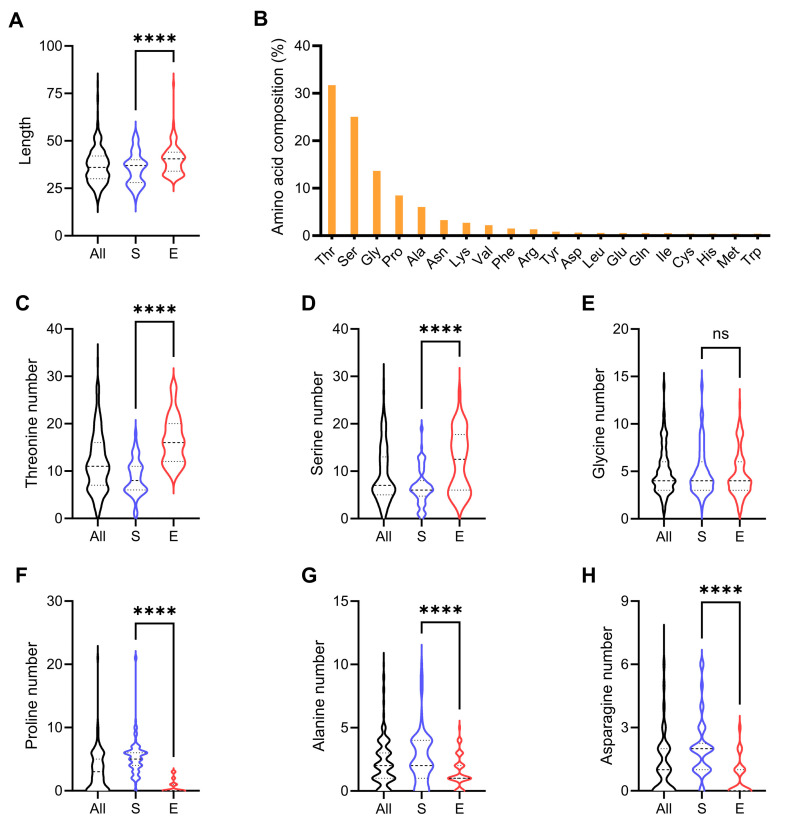
Diversity of linker sequences in GH7 cellobiohydrolases. All: entire dataset (n = 245); S: Sordariomycetes (n = 98); E: Eurotiomycetes (n = 96). (**A**) Amino acid lengths of the linkers. (**B**) Amino acid compositions of all linker sequences. (**C**–**H**) Numbers of the six dominant amino acids in linker sequences. The median and quartile values and statistical significance of the difference between Sordariomycetes and Eurotiomycetes are shown. ns (not significant, *p* > 0.05), **** *p* < 0.0001.

In contrast to the CBM1 domain, the linker region of GH7 cellobiohydrolases does not contain a conserved sequence. Interestingly, the linker sequences of Sordariomycetes and Eurotiomycetes vary significantly in length and amino acid composition. Sordariomycetes cellobiohydrolases generally harbored shorter linker regions than those from Eurotiomycetes (Figure 2A). Further counting of the six most abundant amino acids in the linker sequences suggested a distinct composition of this region between the two classes (Figure 2B–H). Specifically, the linker regions of Sordariomycetes cellobiohydrolases contained less threonine and serine, but more proline, alanine, and asparagine than those from Eurotiomycetes. Consistent with a previous report [13], most of the asparagine residues within the Asn-X-Ser/Thr motifs in the linker sequences (283 of 297) are followed by a proline residue, making them not subject to *N*-glycosylation. Generally, threonine and serine promote flexibility, while proline enforces the rigidity of linker sequences [33], which may consequently affect the hydrolysis performance of the entire enzyme.

### 3.2. Effect of Cel7A-2 Engineering on Cellulase Production in P. oxalicum

**Figure 3:**
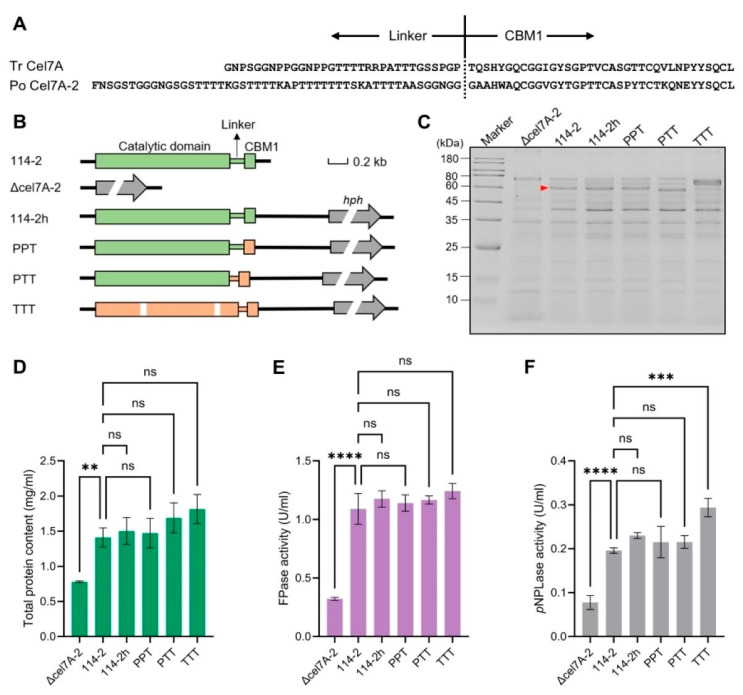
Engineering of *cel7A-2* in *P. oxalicum* via gene replacement. (**A**) Comparison of linker and CBM1 sequences of GH7 cellobiohydrolases from *T. reesei* QM6a and *P. oxalicum* 114-2. (**B**) Schematic illustration of the genetic engineering of *P. oxalicum* 114-2 at the *cel7A-2* locus. The *hph* gene was used as a selection marker. Coding sequences of *P. oxalicum* and *T. reesei* are colored green and orange, respectively. The *T. reesei cel7A* gene contains two introns. (**C**) SDS-PAGE analysis of extracellular proteins at a protein concentration of 0.5 mg/mL. The Cel7A-2 band in 114-2 is indicated by the red arrow. (**D**–**F**) Total protein content, FPase activity, and *p*NPLase activity of the extracellular proteins. The statistical significance of the difference between the 114-2 and mutant strains is shown. ns (not significant, *p* > 0.05), ** *p* < 0.01, *** *p* < 0.001, **** *p* < 0.0001. Data represent the mean ± SD from triplicate cultivations.

Upon cellulose induction, the Eurotiomycete fungus *P. oxalicum* 114-2 secretes two GH7 cellobiohydrolases into the medium, of which Cel7A-2, which harbors a CBM1 domain, is the most abundant cellulase [34]. Previously, we demonstrated that replacing the linker and CBM1 regions of *P. oxalicum* Cel7A-2 with those of *T. reesei* (belonging to the Sordariomycetes class) Cel7A significantly improved its hydrolytic efficiency on microcrystalline cellulose [21] (Figure 3A). However, whether the replacement of the linker region or CBM1 contributed to this improvement remains unknown. Therefore, we engineered the wild-type *P. oxalicum* strain 114-2 to generate three mutant strains that expressed different forms of chimeric GH7 cellobiohydrolase. In the PPT, PTT, and TTT mutants, DNA sequences encoding the CBM1 region, linker-CBM1 region, and entire Cel7A-2 protein were replaced with the corresponding counterparts derived from *T. reesei* (Figure 3B). In addition, the *cel7A-2* gene was deleted to generate the strain Δ*cel7A-2*. As a control, the selection marker gene *hph* used for gene targeting was integrated downstream of the *cel7A-2* coding sequence to generate the strain 114-2h. This allowed us to examine the possible influence of *hph* insertion on the expression of *cel7A-2*.

Culture supernatants of the wild-type strain and five mutants grown in the cellulase production medium were analyzed using SDS-PAGE (Figure 3C). While the protein profiles of 114-2h and PPT were similar to that of 114-2, the band corresponding to Cel7A-2 disappeared in Δ*cel7A-2*. The PTT strain showed a slight downward shift in the Cel7A-2 band, consistent with the shorter linker region in *T. reesei* Cel7A. For strain TTT, replacement of the full *cel7A-2* gene by *T. reesei cel7A* resulted in the detection of a new band in the secretome, while the pattern of the remaining proteins was basically unchanged.

We then compared the concentration and cellulase activity of extracellular proteins among the strains (Figure 3D–F). The Δ*cel7A-2* strain exhibited dramatically lower protein concentration, FPase activity, and *p*NPLase activity than 114-2, revealing the critical role of Cel7A-2 in cellulose degradation. The residual *p*NPLase activity in Δ*cel7A-2* was likely because of the presence of other cellobiohydrolases (e.g., CBM1-lacking Cel7A-1). The concentrations of total proteins and FPase activities of the other mutants were similar to those of 114-2 (Figure 3D,E), suggesting that the engineering of Cel7A-2 did not affect its production level. Notably, the extracellular *p*NPLase activity of TTT was significantly higher than 114-2 (Figure 3F), possibly due to the higher activity of the catalytic domain of *T. reesei* Cel7A towards the substrate *p*NPL. The optimal temperatures of the enzymes produced by 114-2 and TTT were identical (Figure S3), while the other enzymatic properties of the enzymes remain to be compared in future research.

### 3.3. Engineering CBM1 and Linker Region of Cel7A-2 Enhances the Degradation Efficiency of P. oxalicum Cellulase System

**Figure 4:**
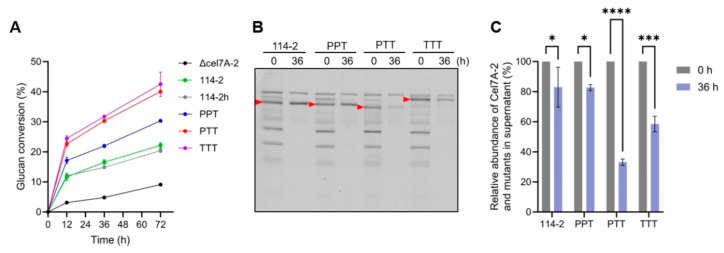
Effect of Cel7A-2 engineering on cellulose hydrolysis efficiency and enzyme adsorption. Avicel (5 g/L) was hydrolyzed by enzymes at 50 °C with a protein-to-substrate ratio of 20 mg/g. (**A**) Glucan conversion during the reaction. (**B**) SDS-PAGE analysis of proteins in the supernatants of the hydrolysis reactions. Equal volumes of supernatants were loaded for comparison. Arrows indicate the bands of Cel7A-2 and its mutants. (**C**) Quantitative analysis of the adsorption of Cel7A-2 and its mutants after 36 h of reaction. Statistical significance of the difference between 0 h and 36 h is shown. * *p* < 0.05, *** *p* < 0.001, **** *p* < 0.0001. Data in (**A**,**C**) represent the mean ± SD of triplicate hydrolysis reactions.

The crude enzyme mixtures secreted by 114-2 and the mutant strains were evaluated for their efficiency in cellulose saccharification under the same protein dosage. In the initial experiment, Avicel, a nearly pure cellulose with a concentration of 5 g/L, was used as the substrate. As shown in Figure 4A, the enzymes produced by strains 114-2 and 114-2h achieved glucan conversions of 22.2% and 20.4%, respectively, after 72 h of reaction. After the elimination of Cel7A-2, glucan conversion decreased to 9.1%, confirming the importance of this enzyme in cellulose degradation. The enzymes produced by strain PPT showed a modest improvement in glucan conversion (30.3%) relative to 114-2, whereas those of PTT achieved 40.0%. This result suggests that sequence variations in both CBM1 and the linker region contributed to the difference in degradation efficiency between *P. oxalicum* Cel7A-2 and *T. reesei* Cel7A. The glucan conversion of enzymes produced by strain TTT showed no significant difference compared to that of PTT, indicating that the catalytic domain is not a major determinant of the efficiency difference between the two enzymes.

The non-catalytic region plays an important role in the binding of cellobiohydrolases to the cellulosic substrates. To assess the adsorption of cellulases on Avicel, the supernatants before and after 36 h of the hydrolysis reaction were analyzed by SDS-PAGE (Figure 4B). The intensities of the bands of Cel7A-2 and its variants, which reflect the abundance of unadsorbed proteins, were quantified (Figure 4C). The amounts of Cel7A-2 in 114-2 and its mutant in PPT in the supernatant both showed slight decreases at 36 h relative to 0 h. This result suggests that there is no significant difference in the cellulose-binding capacity between the two CBM1 domains, at least in the context of being connected to the linker peptide of *P. oxalicum* Cel7A-2. Nevertheless, the two CBM1 domains must differ in some unknown properties, which led to the different glucan conversions between the secretomes of 114-2 and PPT (Figure 4A).

The Cel7A-2 mutant in PTT, which carries the linker-CBM1 region from *T. reesei* Cel7A, displayed a marked reduction of 33.1% in the supernatant after 36 h of hydrolysis. The recombinant *T. reesei* Cel7A in TTT exhibited a 58.6% decrease in its concentration in the supernatant. These results suggest that the non-catalytic region of *T. reesei* Cel7A has a stronger cellulose-binding capacity than that of *P. oxalicum* Cel7A-2, and the linker region plays a dominant role in this difference. The linker peptide of *T. reesei* Cel7A was previously demonstrated to bind directly to cellulose [35], and the present study highlights the critical role of the linker in the binding capacity and hydrolytic activity of cellulase toward cellulose. Compared with that of *P. oxalicum* Cel7A-2, the linker peptide of *T. reesei* Cel7A has three features: shorter length, different amino acid composition (less serine and threonine but more proline), and the absence of an *N*-glycosylation site (Figure 3A). Differences in the length and sequence of the linker have been shown to affect the efficiency of *T. reesei* Cel7A through mutagenesis studies [15,16]. Another possibility is that the *N*-linked glycan at Asn470 of the *P. oxalicum* Cel7A-2 linker may be detrimental to the efficient binding and function of the enzyme, which was circumvented after sequence replacement in the PTT strain. Nevertheless, these hypotheses need to be tested by constructing and characterizing Cel7A-2 mutants with more specific mutations.

### 3.4. The Effect of Non-Catalytic Region Engineering of Cel7A-2 Is Dependent on the Concentration and Property of Substrate

The efficiency of cellulases is significantly influenced by the characteristics and concentration of the substrate [36,37,38]. To further compare the hydrolytic performances of Cel7A-2 and its mutants, saccharification experiments were conducted under three distinct conditions (higher solid loading, lower cellulose crystallinity, and natural lignocellulose as substrate) at 50 °C. Given the demonstrated functional equivalence between TTT and PTT in both FPase activity and cellulose saccharification efficiency (Figure 3E and Figure 4A), we focused on the effects of non-catalytic region modifications and did not include TTT in these comparisons.

**Figure 5:**
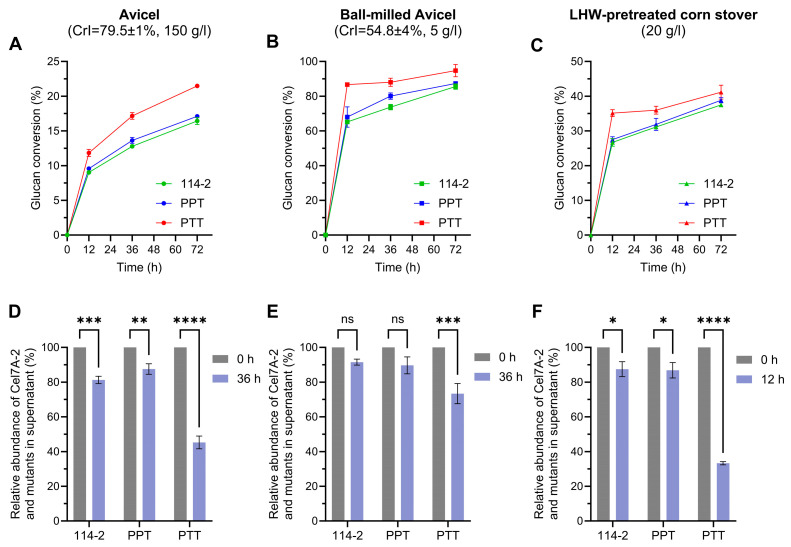
Comparison of the cellulose hydrolytic efficiencies of crude enzymes under different conditions. The reactions were performed at 50 °C. (**A**,**D**) Hydrolysis of 150 g/L Avicel with an enzyme dosage of 4 mg/g substrate. (**B**,**E**) Hydrolysis of 5 g/L ball-milled Avicel with an enzyme dosage of 20 mg/g substrate. (**C**,**F**) Hydrolysis of 20 g/L LHW-pretreated corn stover with an enzyme dosage of 20 mg/g substrate. Glucan conversions (**A**–**C**) and relative abundances of free Cel7A-2 and its mutants in the supernatants (**D**–**F**) are shown. ns (not significant, *p* > 0.05), * *p* < 0.05, ** *p* < 0.01, *** *p* < 0.001, **** *p* < 0.0001. Data are presented as mean ± SD of triplicate hydrolysis reactions.

For the hydrolysis of 150 g/L Avicel, 114-2 and PPT showed similar performances, confirming that the role of CBM1 is less important during the hydrolysis of high-solid substrates [38,39]. Nevertheless, the PTT enzymes demonstrated significantly higher glucan conversion than 114-2 (Figure 5A). For the hydrolysis of 5 g/L ball-milled Avicel with lower crystallinity (Figure S4), the enzymes produced by all strains exhibited substantially elevated glucan conversion compared to untreated Avicel (Figure 4A). While the enzymes of PTT maintained their hydrolytic superiority, inter-strain differences became smaller compared to those on untreated Avicel (Figure 5B). A similar result was observed for the degradation of pretreated corn stover, with PTT enzymes showing higher glucan conversion at the early stages of saccharification (Figure 5C). Protein band analysis revealed stronger adsorption of the Cel7A-2 mutant in PTT onto the substrate relative to 114-2 and PPT (Figure 5D–F), which was consistent with the difference in their hydrolysis efficiencies. Collectively, these results suggest that the linker region of *T. reesei* Cel7A improved the adsorption and degradation activity of *P. oxalicum* Cel7A-2 on cellulosic substrates under various conditions.

### 3.5. Engineering of Non-Catalytic Region of Cel7A-2 Improves Cell Growth on Cellulose

**Figure 6:**
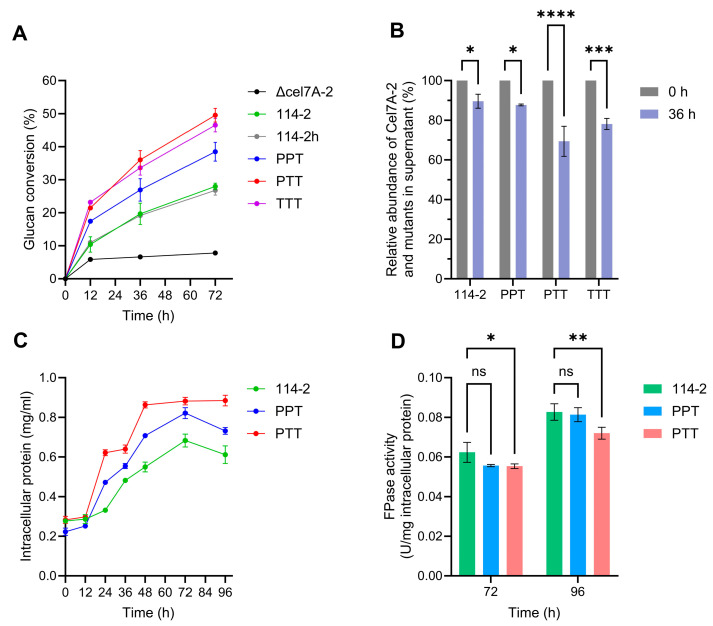
Effect of engineering the catalytic region of Cel7A-2 on cellulose hydrolysis and cell growth at 30 °C. (**A**) Glucan conversion during the hydrolysis of 5 g/L Avicel at 30 °C with a protein-to-substrate ratio of 20 mg/g. (**B**) Relative abundance of free Cel7A-2 and its mutants in the supernatants in the experiment shown in (**A**). (**C**) Intracellular protein content of strains cultured in liquid medium with 5 g/L Avicel as the carbon source at 30 °C. (**D**) Extracellular FPase activities normalized by intracellular protein content in the experiment shown in (**C**). ns (not significant, *p* > 0.05), * *p* < 0.05, ** *p* < 0.01, *** *p* < 0.001, **** *p* < 0.0001. Data represent mean ± SD of triplicate hydrolysis reactions (**A**,**B**) or triplicate cultivations (**C**,**D**).

While all the above hydrolysis experiments were conducted at 50 °C, an industrial conventional temperature for enzymatic saccharification of lignocellulose, the optimal temperature for the growth of *P. oxalicum* was 30 °C. To evaluate the physiological effect of the engineering of the non-catalytic region of Cel7A-2, the crude enzymes produced by 114-2 and engineered strains were used for the hydrolysis of Avicel at 30 °C, with the substrate concentration and enzyme dosage maintained as described in Section 3.3. After 72 h of hydrolysis, the crude enzymes produced by Δ*cel7A-2* exhibited a glucan conversion of 7.8%, whereas the parental strain 114-2 and 114-2h showed comparable conversion values (27.9% and 26.8%, respectively, Figure 6A). The PPT enzymes demonstrated a moderate improvement (38.5%), while the PTT and TTT strains achieved significantly higher conversions (49.6% and 46.5%, respectively). Protein band analysis again revealed stronger adsorption of Cel7A-2 mutants in PTT and TTT to the substrate than those in 114-2 and PPT (Figure 6B). These results suggest that the non-catalytic region of *T. reesei* Cel7A is superior to that of *P. oxalicum* Cel7A-2 at physiological temperatures. Interestingly, all enzyme systems, except Δ*cel7A-2*, achieved higher glucan conversions at 72 h at 30 °C than at 50 °C (Figure 4A), the reason for which remains to be determined.

To further assess the impact of the engineering of the non-catalytic region of Cel7A-2 on cellulose-dependent growth, strains 114-2, PPT, and PTT were cultivated in liquid medium with Avicel as the sole carbon source. The measurement of intracellular protein content, representing cell biomass, showed that PTT exhibited the best growth on Avicel, followed by PPT and 114-2 (Figure 6C). The biomass of 114-2 and PPT declined after 72 h, likely due to autolysis under prolonged cultivation, whereas PTT maintained a stable biomass from 72 h to 96 h. When the extracellular FPase activity was normalized by the intracellular protein content, PPT and PTT showed no superiority over 114-2 in cellulase production (Figure 6D), suggesting that the better growth of *cel7A-2*-engineered strains was mainly caused by the higher efficiency, but not by higher production of their cellulase systems.

## 4. Conclusions

In this study, we demonstrated that the inter-domain linker region of GH7 cellobiohydrolases from Sordariomycetes is statistically longer and contains less threonine and serine and more proline than that from Eurotiomycetes. Through the sequence replacement of *P. oxalicum* Cel7A-2 by the counterparts in *T. reesei* Cel7A, we showed that both the cellulose-binding domain and linker peptide are responsible for the higher efficiency of the latter enzyme in the hydrolysis of crystalline cellulose. In addition, growth tests indicated that the sequence variation in the non-catalytic region of GH7 cellobiohydrolases may affect the fitness of cellulolytic fungi in natural environments. Given the widespread occurrence of the catalytic domain-linker-CBM architecture in biopolymer-degrading enzymes, our findings highlight the linker-CBM region as a functionally tunable module that likely drives adaptive evolution in these enzymes. This work challenges the traditional catalytic domain-centric engineering paradigm and proposes a shift toward non-catalytic optimization strategies in cellulase design.

## Data Availability

The data supporting the results reported in this study are included in the article/Supplementary Materials. Further inquiries should be directed to the corresponding author.

## Supplementary Materials

The following supporting information can be downloaded at: https://www.mdpi.com/article/10.3390/jof11070536/s1. Figure S1. Phylogenetic analysis of CBM1 sequences of 245 CBH Is in this study. Of the bootstrap values obtained, 77.0% showed significant support (>50%). Sequences from Sordariomycetes, Eurotiomycetes, and other fungi are indicated by blue, red, and yellow backgrounds, respectively. Figure S2. Phylogenetic analysis of the catalytic domain sequences of 245 CBH Is in this study. Of the bootstrap values obtained, 96.7% showed significant support (>50%). Sequences from Sordariomycetes, Eurotiomycetes, and other fungi are indicated by blue, red, and yellow backgrounds, respectively. Figure S3. FPase and *p*NPLase activities of 114-2 and TTT enzymes at different temperatures. Figure S4. X-ray diffraction analysis of untreated and ball-milled Avicel. X-ray diffraction data were collected with the intensity expressed in Counts Per Second (CPS). Table S1. Primers used for constructing *cel7A-2*-engineered strains. Table S2. GH7 cellobiohydrolases analyzed in this study.

## References

[1] Cragg S.M., Beckham G.T., Bruce N.C., Bugg T.D.H., Distel D.L., Dupree P., Etxabe A.G., Goodell B.S., Jellison J., McGeehan J.E. (2015). Lignocellulose degradation mechanisms across the tree of life. Curr. Opin. Chem. Biol..

[2] Chen C.-C., Dai L., Ma L., Guo R.-T. (2020). Enzymatic degradation of plant biomass and synthetic polymers. Nat. Rev. Chem..

[3] Chundawat S.P.S., Beckham G.T., Himmel M.E., Dale B.E. (2011). Deconstruction of lignocellulosic biomass to fuels and chemicals. Annu. Rev. Chem. Biomol. Eng..

[4] Su C., Cai D., Zhang H., Wu Y., Jiang Y., Liu Y., Zhang C., Li C., Qin P., Tan T. (2024). Pilot-scale acetone-butanol-ethanol fermentation from corn stover. Green Carbon.

[5] Li Z., Waghmare P.R., Dijkhuizen L., Meng X., Liu W. (2024). Research advances on the consolidated bioprocessing of lignocellulosic biomass. Eng. Microbiol..

[6] Teeri T.T., Koivula A., Linder M., Wohlfahrt G., Divne C., Jones T.A. (1998). *Trichoderma reesei* cellobiohydrolases: Why so efficient on crystalline cellulose?. Biochem. Soc. Trans..

[7] Ren M., Wang Y., Liu G., Zuo B., Zhang Y., Wang Y., Liu W., Liu X., Zhong Y. (2020). The effects of deletion of cellobiohydrolase genes on carbon source-dependent growth and enzymatic lignocellulose hydrolysis in *Trichoderma reesei*. J. Microbiol..

[8] Lombard V., Golaconda Ramulu H., Drula E., Coutinho P.M., Henrissat B. (2014). The carbohydrate-active enzymes database (CAZy) in 2013. Nucleic Acids Res..

[9] Igarashi K., Koivula A., Wada M., Kimura S., Penttilä M., Samejima M. (2009). High speed atomic force microscopy visualizes processive movement of *Trichoderma reesei* cellobiohydrolase I on crystalline cellulose. J. Biol. Chem..

[10] Liu Y.-S., Baker J.O., Zeng Y., Himmel M.E., Haas T., Ding S.-Y. (2011). Cellobiohydrolase hydrolyzes crystalline cellulose on hydrophobic faces. J. Biol. Chem..

[11] Li S., Liu G. (2024). Harnessing cellulose-binding protein domains for the development of functionalized cellulose materials. Bioresour. Bioprocess..

[12] Mello B., Polikarpov I. (2014). Family 1 carbohydrate binding-modules enhance saccharification rates. AMB Express.

[13] Sammond D.W., Payne C.M., Brunecky R., Himmel M.E., Crowley M.F., Beckham G.T. (2012). Cellulase linkers are optimized based on domain type and function: Insights from sequence analysis, biophysical measurements, and molecular simulation. PLoS ONE.

[14] Reinikainen T., Ruohonen L., Nevanen T., Laaksonen L., Teeri T.T. (1992). Investigation of the function of mutated cellulose-binding domains of *Trichoderma reesei* cellobiohydrolase I. Proteins.

[15] Srisodsuk M., Reinikainen T., Penttil M., Teeri T.T. (1993). Role of the interdomain linker peptide of *Trichoderma reesei* cellobiohydrolase i in its interaction with crystalline cellulose. J. Biol. Chem..

[16] Badino S.F., Bathke J.K., Sørensen T.H., Windahl M.S., Jensen K., Peters G.H.J., Borch K., Westh P. (2017). The influence of different linker modifications on the catalytic activity and cellulose affinity of cellobiohydrolase *cel7a* from *Hypocrea jecorina*. Protein Eng. Des. Sel..

[17] Voutilainen S.P., Puranen T., Siika-aho M., Lappalainen A., Alapuranen M., Kallio J., Hooman S., Viikari L., Vehmaanperä J., Koivula A. (2008). Cloning, expression, and characterization of novel thermostable family 7 cellobiohydrolases. Biotechnol. Bioeng..

[18] Taylor L.E., Knott B.C., Baker J.O., Alahuhta P.M., Hobdey S.E., Linger J.G., Lunin V.V., Amore A., Subramanian V., Podkaminer K. (2018). Engineering enhanced cellobiohydrolase activity. Nat. Commun..

[19] Brunecky R., Knott B.C., Subramanian V., Linger J.G., Beckham G.T., Amore A., Taylor L.E., Vander Wall T.A., Lunin V.V., Zheng F. (2024). Engineering of glycoside hydrolase family 7 cellobiohydrolases directed by natural diversity screening. J. Biol. Chem..

[20] Xue J., Jiang X., Li A., Li J., Su X., Huang J., Qin L. (2025). Catalytic efficiency improvement in cellobiohydrolase I by cross-species domain exchange engineering. Int. J. Mol. Sci..

[21] Du J., Zhang X., Li X., Zhao J., Liu G., Gao B., Qu Y. (2018). The cellulose binding region in *Trichoderma reesei* cellobiohydrolase I has a higher capacity in improving crystalline cellulose degradation than that of *Penicillium oxalicum*. Bioresour. Technol..

[22] Madeira F., Pearce M., Tivey A.R.N., Basutkar P., Lee J., Edbali O., Madhusoodanan N., Kolesnikov A., Lopez R. (2022). Search and sequence analysis tools services from EMBL-EBI in 2022. Nucleic Acids Res..

[23] Gado J.E., Harrison B.E., Sandgren M., Ståhlberg J., Beckham G.T., Payne C.M. (2021). Machine learning reveals sequence-function relationships in family 7 glycoside hydrolases. J. Biol. Chem..

[24] Crooks G.E., Hon G., Chandonia J.M., Brenner S.E. (2004). Weblogo: A sequence logo generator. Genome Res..

[25] Nguyen L.-T., Schmidt H.A., von Haeseler A., Minh B.Q. (2015). IQ-TREE: A fast and effective stochastic algorithm for estimating maximum-likelihood phylogenies. Mol. Biol. Evol..

[26] Letunic I., Bork P. (2021). Interactive tree of life (iTOL) v5: An online tool for phylogenetic tree display and annotation. Nucleic Acids Res..

[27] Liu G., Qin Y., Li Z., Qu Y. (2014). Improving lignocellulolytic enzyme production with *Penicillium*: From strain screening to systems biology. Biofuels.

[28] Gao L., Li Z., Xia C., Qu Y., Liu M., Yang P., Yu L., Song X. (2017). Combining manipulation of transcription factors and overexpression of the target genes to enhance lignocellulolytic enzyme production in *Penicillium oxalicum*. Biotechnol. Biofuels.

[29] Vogel H.J.A. (1956). A convenient growth medium for neurospora (medium V). Microb. Genet. Bull..

[30] Schneider C.A., Rasband W.S., Eliceiri K.W. (2012). NIH image to ImageJ: 25 years of image analysis. Nat. Methods.

[31] Rueden C.T., Schindelin J., Hiner M.C., DeZonia B.E., Walter A.E., Arena E.T., Eliceiri K.W. (2017). ImageJ2: ImageJ for the next generation of scientific image data. BMC Bioinform..

[32] Salem K.S., Kasera N.K., Rahman M.A., Jameel H., Habibi Y., Eichhorn S.J., French A.D., Pal L., Lucia L.A. (2023). Comparison and assessment of methods for cellulose crystallinity determination. Chem. Soc. Rev..

[33] Chen X., Zaro J.L., Shen W.-C. (2013). Fusion protein linkers: Property, design and functionality. Adv. Drug Deliv. Rev..

[34] Liu G., Zhang L., Wei X., Zou G., Qin Y., Ma L., Li J., Zheng H., Wang S., Wang C. (2013). Genomic and secretomic analyses reveal unique features of the lignocellulolytic enzyme system of *Penicillium decumbens*. PLoS ONE.

[35] Payne C.M., Resch M.G., Chen L., Crowley M.F., Himmel M.E., Taylor L.E., Sandgren M., Ståhlberg J., Stals I., Tan Z. (2013). Glycosylated linkers in multimodular lignocellulose-degrading enzymes dynamically bind to cellulose. Proc. Natl. Acad. Sci. USA.

[36] Chundawat S.P.S., Bellesia G., Uppugundla N., da Costa Sousa L., Gao D., Cheh A.M., Agarwal U.P., Bianchetti C.M., Phillips G.N., Langan P. (2011). Restructuring the crystalline cellulose hydrogen bond network enhances its depolymerization rate. J. Am. Chem. Soc..

[37] Shi R., Zhang Z., Zhang J., Chen C., Li W., Lin Y., Shi X., Zhao P., Zhang T., Yan Q. (2025). A comparative study on enhanced enzymatic hydrolysis of diverse herbaceous and woody wastes by promising dilute acid and alkaline pretreatments. Bioresour. Bioprocess..

[38] Le Costaouëc T., Pakarinen A., Várnai A., Puranen T., Viikari L. (2013). The role of carbohydrate binding module (CBM) at high substrate consistency: Comparison of *Trichoderma reesei* and *Thermoascus aurantiacus* Cel7A (CBHI) and Cel5A (EGII). Bioresour. Technol..

[39] Pakarinen A., Haven M., Djajadi D., Várnai A., Puranen T., Viikari L. (2014). Cellulases without carbohydrate-binding modules in high consistency ethanol production process. Biotechnol. Biofuels.

