# Supplementary material for: The Role of Non-Catalytic Region in Determining the Difference in Efficiency Between Two Cellobiohydrolases Revealed Through a Genetic Approach
Source: J Fungi (Basel). 2025 Jul 18;11(7):536. doi: 10.3390/jof11070536 (PMC12299840; doi:10.3390/jof11070536)
Supplement: Supplementary file 1 [file jof-11-00536-s001.zip › Figure S4 X-ray diffraction analysis of untreated and ball-milled Avicel.pdf]

Journal of Fungi

MDPI

Figure S4. X-ray diffraction analysis of untreated and ball-milled Avicel. X-ray diffraction data were collected with intensity expressed in Counts Per Second (CPS).

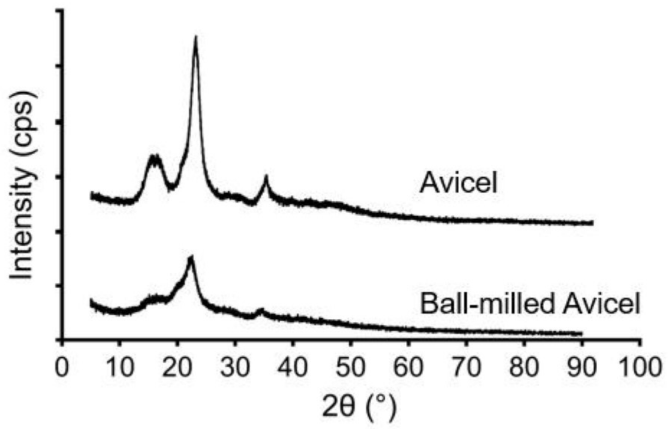

 https://doi.org/10.3390/xxxxx
