# Supplementary material for: The Role of Non-Catalytic Region in Determining the Difference in Efficiency Between Two Cellobiohydrolases Revealed Through a Genetic Approach
Source: J Fungi (Basel). 2025 Jul 18;11(7):536. doi: 10.3390/jof11070536 (PMC12299840; doi:10.3390/jof11070536)
Supplement: Supplementary file 1 [file jof-11-00536-s001.zip › Table S1 Primers used for the construction of cel7A-2-engineered strains.pdf]

Table S1. Primers used for the construction of *cel7A-2*-engineered strains.

Name	Sequence (5'→3')	Product
114-2h-Up-F	TGCCCTCTACTTCGTCGACATGG	Left arm for the construction of 114-2h
114-2h-Uphph-R	TTCAATATCAGTTAACGTCGACGCCAGTC CAGTGATTAGGTG	
hph-F-c	CCTAATCACTGGACTGGCGTCGACGTTAA CTGATATTGAAG	*hph* as a selection marker for the construction of all mutant strains except Δ*cel7A-2*
hph-R-c	CCAGTGTGTACACAACCTGGCAACCCAGG GCTGGTGACGG	
114-2h-Dnhph-F	CCGTCACCAGCCCTGGGTTGCCAGGTTGT GTACACACTGGAG	Right arm for the construction of 114-2h
114-2h-Dn-R	CAGTCCACGAGATTGCAGGTCG	
iC-Up-F	CGATGCGCCAAAGACAGTCATTG	Left arm for the construction of Δ*cel7A-2*
iC-Uphph-R	CCTTCAATATCAGTTAACGTCGTGTGATGG ATTGGATCAAAGATC	
iC-hph-Dn-F	GATCTTTGATCCAATCCATCACACGACGTT AACTGATATTGAAGG	*hph* + right arm for the construction of Δ*cel7A-2*
iC-hph-Dn-R	CCGTTGATGCGAGGCTTACATTG	
Up-TCBHI-F	CGAACTTCCATTGATCTGCCCG	Left arm for the construction of TTT
Up-TCBHI-R	GACGGCCAACTTCCGATACATTGTGATGG ATTGGATCAAAGATC	
TCBHI-Up-F	GATCTTTGATCCAATCCATCACAATGTATC GGAAGTTGGCCGTC	*T. reesei cel7A* for the construction of TTT
TCBM1-hph-R	CGACGACCACTCAAGACTTACAGGCACTG AGAGTAGTAAG	
T-hph-Dn-F	CTTACTACTCTCAGTGCCTGTAAGTCTTGA GTGGTCGTCG	Terminator + *hph* + right arm for the construction of TTT
T-hph-Dn-R	ATCGGGCTCCTTGAGAAGATGC	
PPT/PTT-Up-F	ATGAAGGGTTCCATCTCCTACC	See below
PPT-UpCBM1-R	TGGCCGTAGTGAGACTGGGTGCCGCCGTT ACCGCCAGAGG	Left arm for the construction of PPT (with PPT/PTT-Up-F)
PTT-Uplinker-R	TTGCCGCCGCTAGGGTTGCCGGTGGAGCC AATGGGACCAG	Left arm for the construction of PTT (with PPT/PTT-Up-F)
PPT-CBM1Dn-F	CCTCTGGCGGTAACGGCGGCACCCAGTCT CACTACGGCCA	*T. reesei cel7A* CBM1 + terminator + *hph* + right arm for the construction of PPT (with T-hph-Dn-R)
PTT-linkerDn-F	CTGGTCCCATTGGCTCCACCGGCAACCCT AGCGGCGGCAA	*T. reesei cel7A* linker-CBM1 + terminator + *hph* + right arm for the construction of PTT (with T-hph-Dn-R)
