# Supplementary figures and images for: The Role of Non-Catalytic Region in Determining the Difference in Efficiency Between Two Cellobiohydrolases Revealed Through a Genetic Approach
Source: J Fungi (Basel). 2025 Jul 18;11(7):536. doi: 10.3390/jof11070536 (PMC12299840; doi:10.3390/jof11070536)

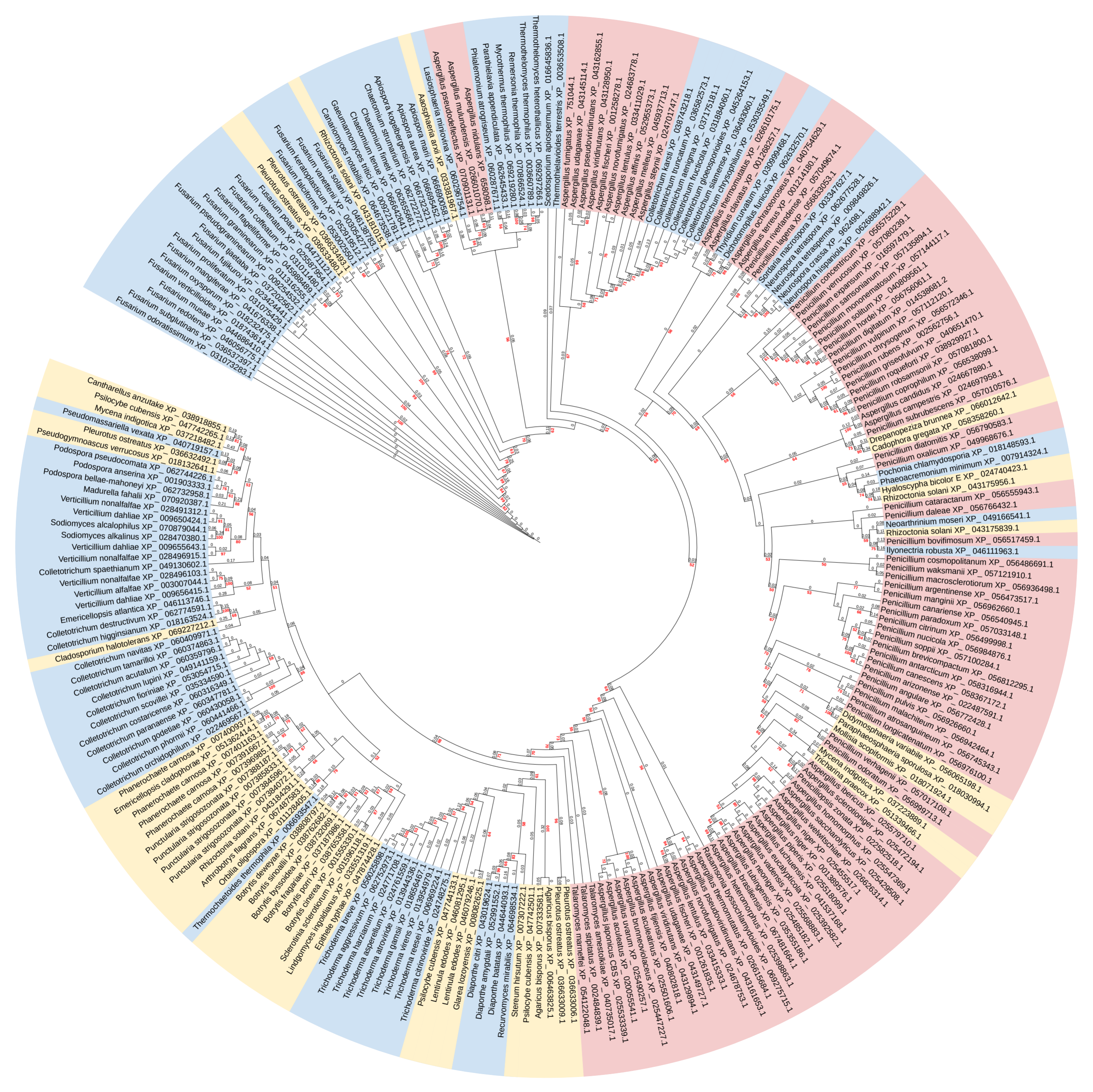

Supplement: Supplementary file 1 [file jof-11-00536-s001.zip › Figure S1 Phylogenetic analysis of CBM1 sequences of 245 CBH Is in this study.pdf]

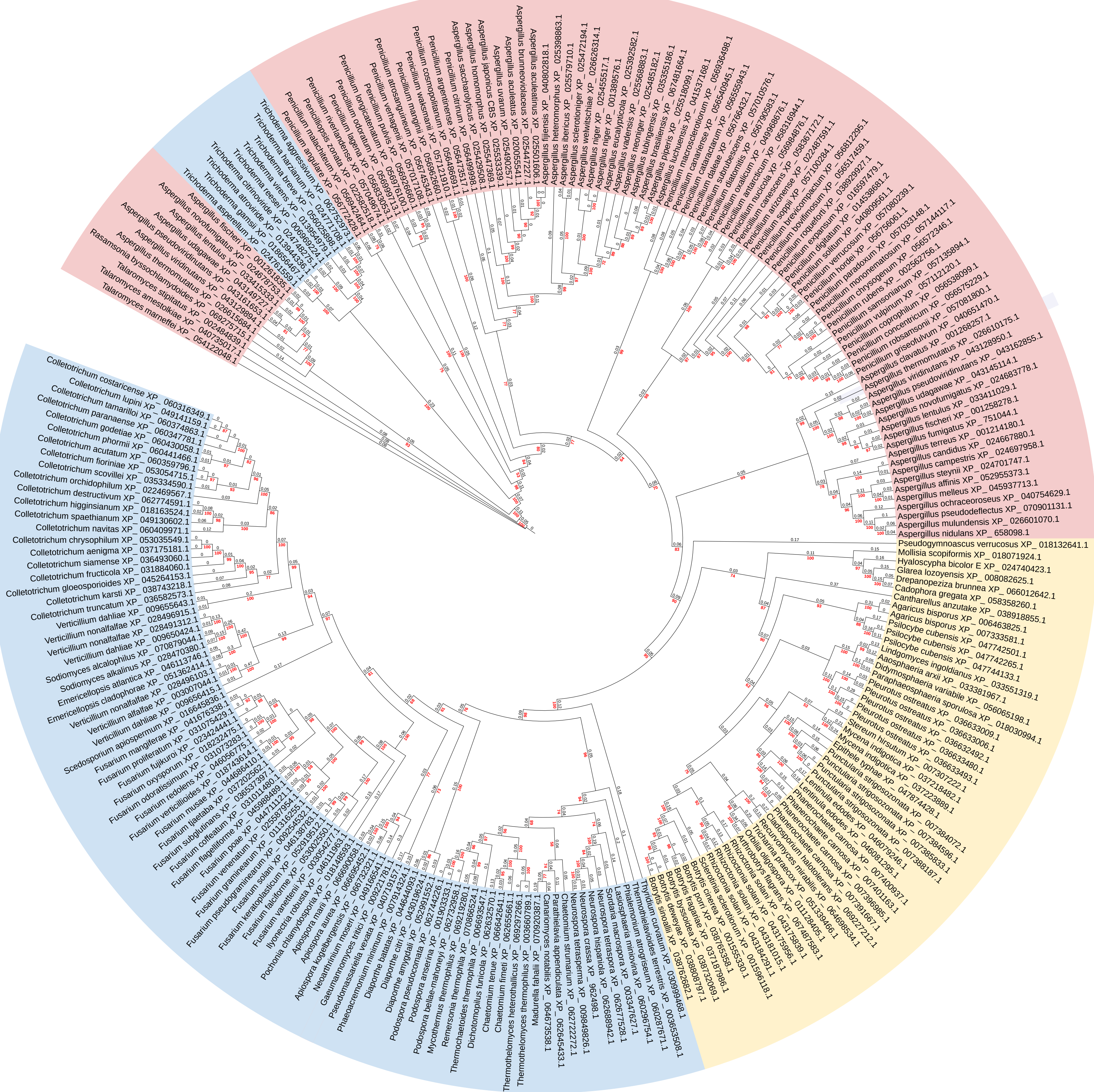

Supplement: Supplementary file 1 [file jof-11-00536-s001.zip › Figure S2 Phylogenetic analysis of catalytic domain sequences of 245 CBH Is in this study.pdf]

**Figure S3.** FPase and *p*NPLase activities of 114-2 and TTT enzymes at different temperatures.

1

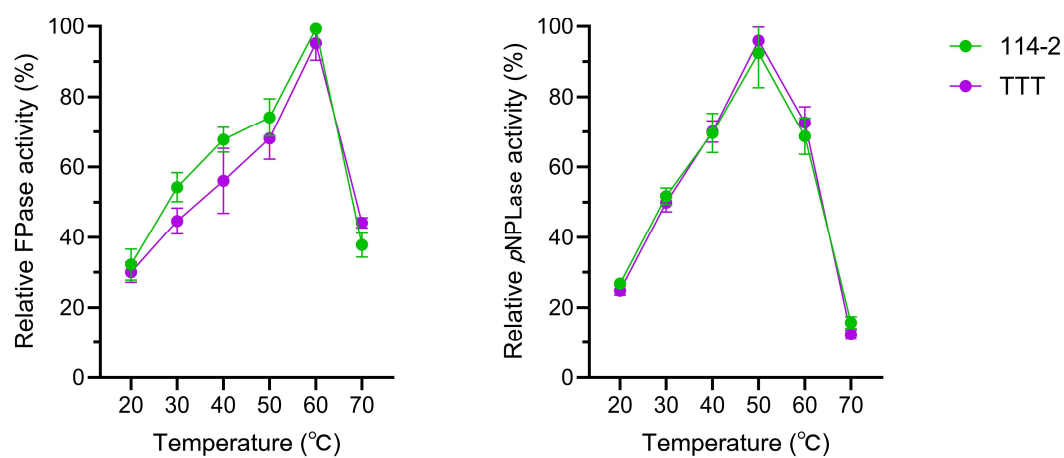

2

Supplement: Supplementary file 1 [file jof-11-00536-s001.zip › Figure S3 FPase and pNPLase activities of 114-2 and TTT enzymes at different temperatures.pdf]
